# New evidence of a mitochondrial genetic background paradox: Impact of the J haplogroup on the A3243G mutation

**DOI:** 10.1186/1471-2350-9-41

**Published:** 2008-05-07

**Authors:** Denis Pierron, Christophe Rocher, Patricia Amati-Bonneau, Pascal Reynier, Marie-Laure Martin-Négrier, Stéphane Allouche, Cécile Batandier, Benedicte Mousson de Camaret, Catherine Godinot, Agnes Rotig, Delphine Feldmann, Christine Bellanne-Chantelot, Benoit Arveiler, Erwann Pennarun, Rodrigue Rossignol, Marc Crouzet, Pascal Murail, Didier Thoraval, Thierry Letellier

**Affiliations:** 1 Université Bordeaux 1, Laboratoire d'Anthropologie des Populations du Passé, UMR 5199 PACEA, 33400 Talence, France; 2 Institut de Biochimie et Génétique Cellulaires, UMR 5095, CNRS, Université Victor Segalen-Bordeaux 2, 33076 Bordeaux, France; 3 INSERM, U688 Laboratoire de Physiopathologie Mitochondriale; Université Victor Segalen-Bordeaux 2, 33076 Bordeaux, France; 4 INSERM, U694, Angers, F-49033 France; Departement de Biochimie et Génétique, Centre Hospitalier Universitaire, Angers, F-49033 France; 5 Laboratoire d'Anatomie Pathologique, Centre Hospitalier Universitaire, Laboratoire d'Histologie-Embryologie, UFRII, Université Victor Segalen Bordeaux 2, 33076 Bordeaux, France; 6 Laboratoire de Biologie cellulaire et moléculaire de la signalisation, UPRES-EA 3919, Université de Caen, 14033 Caen, France; 7 Laboratoire de Biochimie et Génétique Moléculaire, Centre Hospitalier Universitaire de Grenoble, 38054 Grenoble, France; 8 Laboratoire de Biochimie Pédiatrique, Hôpital Debrousse, 69322 Lyon, France; 9 Centre de Génétique Moléculaire et Cellulaire, UMR 5534, CNRS, Université Claude Bernard de Lyon 1, 69622 Villeurbanne, France; 10 Department of Genetics, INSERM U781 Hôpital Necker-Enfants Malades, 75743 Paris, France; 11 Service de Biochimie et de Biologie Moléculaire, INSERM U587 Hôpital d'Enfants Armand-Trousseau, AP-HP, 75571 Paris, France; 12 Department of Cytogenetics, Hôpital Saint-Antoine-AP-HP, 75012 Paris, France; 13 Pôle Génotypage-Séquençage, Université Victor Segalen Bordeaux 2, 33076 Bordeaux, France; 14 Department of Evolutionary Biology, Institute of Molecular and Cell Biology, University of Tartu and Estonian Biocentre, Tartu, Estonia

## Abstract

**Background:**

The A3243G mutation in the tRNALeu gene (UUR), is one of the most common pathogenic mitochondrial DNA (mtDNA) mutations in France, and is associated with highly variable and heterogeneous disease phenotypes. To define the relationships between the A3243G mutation and mtDNA backgrounds, we determined the haplogroup affiliation of 142 unrelated French patients – diagnosed as carriers of the A3243G mutation – by control-region sequencing and RFLP survey of their mtDNAs.

**Results:**

The analysis revealed 111 different haplotypes encompassing all European haplogroups, indicating that the 3243 site might be a mutational hot spot. However, contrary to previous findings, we observed a statistically significant underepresentation of the A3243G mutation on haplogroup J in patients (p = 0.01, OR = 0.26, C.I. 95%: 0.08–0.83), suggesting that might be due to a strong negative selection at the embryo or germ line stages.

**Conclusion:**

Thus, our study supports the existence of mutational hotspot on mtDNA and a "haplogroup J paradox," a haplogroup that may increase the expression of mtDNA pathogenic mutations, but also be beneficial in certain environmental contexts.

## Background

The mitochondrion is unique in that its bioenergetic and biosynthetic systems are assembled from two different information storage and retrieval systems: the nuclear-cytosolic system, expressing nuclear DNA-encoded mitochondrial genes, and the mitochondrial system, expressing mitochondrial DNA-encoded genes. Maternally inherited, mitochondrial DNA (mtDNA) encodes the 12S and 16S mitochondrial rRNAs, 22 tRNAs, and 13 polypeptides, which form essential subunits of OXPHOS enzyme complexes [1,2].

Over the past twenty years, genetic variations in mtDNA have been linked to a number of multifactorial diseases and aging [3], however, a large number of mutations were considered neutral and used to define haplogroups in order to investigate human origins [4], migrations, and demographic events [5]. Indeed, human populations can be divided into several mtDNA haplogroups on the basis of specific nucleotid polymorphisms scattered throughout the mitochondrial genome, reflecting mutation events accumulated over time by a discrete maternal lineage. Thus, for example, 95% of Europeans belong to 10 major haplogroups [6] while 100% of Native Americans belong to 5 major haplogroups [7,8].

Several recent studies have pointed out the possible impact of haplogroups on mitochondrial physiology. Indeed, Ruiz-Pesini et al. [9] revealed a possible impact of haplogroups on the oxidative phosphorylation activity that facilitates human adaptation to colder climates, resulting in regional enrichment of specific mtDNA lineages (haplogroups). Moreover, haplogroups have been shown to play a role in the phenotypic expression of diseases [10]. Indeed, a link has been observed between haplogroups and various disorders and phenotypes; for example, the haplogroup may influence the disease expression of a mitochondrial pathogenic mutation, such as T14484C and G11778A, found in Leber hereditary optic neuropathy (LHON) [11-15].

The A3243G mutation [16-18] on the tRNALeu gene (UUR) is one of the most common mitochondrial DNA mutations worldwide and in France (unpublished data from the French mitochondrial disease network). The phenotypic expression of this mutation is quite variable, ranging from mild to severe clinical phenotypes. Indeed, while mtDNA the A3243G mutation is usually associated with the MELAS syndrome (mitochondrial myopathy, encephalopathy, lactic acidosis, and strokelike episodes: MIM 540000) or MIDD (maternally inherited diabetes and deafness: MIM 520000) [19]. This mutation is also found in patients without any typical symptoms, but who had clinical manifestations ranging from a mixture of chronic progressive external ophthalmoplegia symptoms, to strokelike episodes, cardiomyopathy, and progressive kidney disease [20-23].

Several molecular mechanisms have been proposed to explain the phenotypic expression of this mutation, such as a reduced association of mRNA with ribosomes, possibly due to the tRNALeu (UUR) aminoacylation defect [24]. However, Kirino et al. recently proposed that a wobble modification deficiency resulting in defective translation could be a key molecular factor responsible for the phenotypic features of MELAS that would explain why the different MELAS-associated mutations produce indistinguishable clinical features [25].

Several studies suggested that certain mtDNA haplogroups affected the phenotypic expression of this mutation [26,27], but Torroni et al. and Deschauer et al. recently concluded that European mtDNA backgrounds did not have any substantial impact [28,29], however, these studies involved only a small number of individuals or poorly-defined populations. Thus it was impossible to confirm the relationship between mutations responsible for pathological conditions and haplogroups. Indeed, as mentioned by Samuels et al. [30], large cohorts are required to identify a reliable connection with complex human diseases and geographical variations in haplogroup frequencies.

In order to elucidate the relationships between mtDNA the A3243G mutation and mitochondrial genetic backgrounds, we used RFLP and sequencing to determine haplogroups of 142 patients diagnosed as carriers of the A3243G mutation by the French mitochondrial disease network.

## Methods

### A3243G patients

Screening for mitochondrial diseases was carried out by the French mitochondrial disease network. This network is made up of all the laboratories involved in genetic and biochemical diagnosis of mitochondrial pathologies in France. These laboratories collect samples from all French patients suspected to be affected by mitochondrial diseases, as the French health-care system makes it possible for each individual to receive appropriate treatment. Over the past fifteen years, 1,667 patients have been diagnosed as carriers of a mitochondrial mutation. Patients showing symptoms of MELAS, deafness, or mitochondrial diabetes were tested for the A3243G mutation, using standard procedures. Among the cohort of individuals diagnosed as carriers of the A3243G mutation over this period, we have listed 142 unrelated patients tested by laboratories in University Hospitals of Angers, Bordeaux, Caen, Grenoble, Lyon, and Paris. All these patients carried the A3243G mutation and presented a mitochondrial pathology (General phenotype are presented in Table 1). Despite we have detected qualitatively for all the A3243G patients the presence of heteroplamy (between 80 to 40%), the precise percentage of heteroplasmy was not available for all the patients. Genetic analyses were carried out with the appropriate consent of the patients.

**Table 1:** RFLP and control-region mtDNA haplotypes from the French A3243G carriers. The patients were regrouped in three major phenotypes (Clear MELAS Syndrome, Partial MELAS syndrome and Diabetes Syndrome).

**Individu**	**haplotype**	**haplogroup**	**RFLP**	**POLYMORPHISM OBSERVED BETWEEN POSITION 16050–16569 AND 1- 205**	**PHENOTYPE**
**1**	**1**	H	-7025AluI	16304,	clear MELAS syndrome
**2**	**1**	H	-7025AluI	16304,	Partial MELAS syndrome
**3**	**1**	H	-7025AluI	16304,	clear MELAS syndrome
**4**	**2**	H	-7025AluI	16354,	Diabetes Syndrome
**5**	**2**	H	-7025AluI	16354,	Diabetes Syndrome
**6**	**3**	H	-7025AluI	16519,	Partial MELAS syndrome
**7**	**3**	H	-7025AluI	16519,	Diabetes Syndrome
**8**	**3**	H	-7025AluI	16519,	clear MELAS syndrome
**9**	**3**	H	-7025AluI	16519,	Diabetes Syndrome
**10**	**3**	H	-7025AluI	16519,	Diabetes Syndrome
**11**	**3**	H	-7025AluI	16519,	clear MELAS syndrome
**12**	**3**	H	-7025AluI	16519,	Partial MELAS syndrome
**13**	**3**	H	-7025AluI	16519,	clear MELAS syndrome
**14**	**3**	H	-7025AluI	16519,	clear MELAS syndrome
**15**	**3**	H	-7025AluI	16519,	Partial MELAS syndrome
**16**	**3**	H	-7025AluI	16519,	Diabetes Syndrome
**17**	**3**	H	-7025AluI	16519,	clear MELAS syndrome
**18**	**3**	H	-7025AluI	16519,	Diabetes Syndrome
**19**	**4**	H	-7025AluI	16094,16278,16293,16311,195,	clear MELAS syndrome
**20**	**5**	H	-7025AluI	16114,16293,16311,16354,16519,195,	clear MELAS syndrome
**21**	**5**	H	-7025AluI	16114,16293,16311,16354,16519,195,	Diabetes Syndrome
**22**	**6**	H	-7025AluI	16126,16274,16519,	Diabetes Syndrome
**23**	**7**	H	-7025AluI	16126,16311,16519,	Diabetes Syndrome
**24**	**8**	H	-7025AluI	16126,16519,198,	clear MELAS syndrome
**25**	**9**	H	-7025AluI	16129,16316,16519,	Partial MELAS syndrome
**26**	**10**	H	-7025AluI	16129,16362,16482,16519,	Partial MELAS syndrome
**27**	**11**	H	-7025AluI	16129,16519,	Diabetes Syndrome
**28**	**12**	H	-7025AluI	16129,16519,10,	Partial MELAS syndrome
**29**	**13**	H	-7025AluI	16129,16519,10,189,	clear MELAS syndrome
**30**	**14**	H	-7025AluI	16129,16519,152,	clear MELAS syndrome
**31**	**15**	H	-7025AluI -12308HinfI; -4216NlaIII; +4577NlaIII	16162,16209,16519,73,	Diabetes Syndrome
**32**	**16**	H	-7025AluI	16166delA,	Diabetes Syndrome
**33**	**17**	H	-7025AluI	16169,16299,16519,	Diabetes Syndrome
**34**	**18**	H	-7025AluI	16183C,16189,16519,	clear MELAS syndrome
**35**	**19**	H	-7025AluI	16189,195,	Partial MELAS syndrome
**36**	**19**	H	-7025AluI	16189,195,	clear MELAS syndrome
**37**	**19**	H	-7025AluI	16189,195,	clear MELAS syndrome
**38**	**20**	H	-7025AluI	16213,16256,16519,9,	Partial MELAS syndrome
**39**	**21**	H	-7025AluI	16221,16519,	Partial MELAS syndrome
**40**	**22**	H	-7025AluI	16260,16357,16519,	Diabetes Syndrome
**41**	**22**	H	-7025AluI	16260,16357,16519,	Partial MELAS syndrome
**42**	**23**	H	-7025AluI	16261,16519,	clear MELAS syndrome
**43**	**24**	H	-7025AluI -12308HinfI; -4216NlaIII; +4577NlaIII	16278,16519,73,	Partial MELAS syndrome
**44**	**25**	H	-7025AluI	16290,16304,16519,	Diabetes Syndrome
**45**	**26**	H	-7025AluI	16290,16519,152,	Diabetes Syndrome
**46**	**27**	H	-7025AluI	16293,16519,	Diabetes Syndrome
**47**	**27**	H	-7025AluI	16293,16519,	Diabetes Syndrome
**48**	**28**	H	-7025AluI	16304,16325,	clear MELAS syndrome
**49**	**29**	H	-7025AluI	16311,16519,	Diabetes Syndrome
**50**	**29**	H	-7025AluI	16311,16519,	Diabetes Syndrome
**51**	**29**	H	-7025AluI	16311,16519,	Diabetes Syndrome
**52**	**30**	H	-7025AluI	16311,16519,93,	Diabetes Syndrome
**53**	**31**	H	-7025AluI	16362,16482,	clear MELAS syndrome
**54**	**32**	H	-7025AluI	16362,16482,16519,152,	Partial MELAS syndrome
**55**	**33**	H	-7025AluI	16519,143,152,	Partial MELAS syndrome
**56**	**34**	H	-7025AluI	16519,151,152,	Partial MELAS syndrome
**57**	**34**	H	-7025AluI	16519,151,152,	Partial MELAS syndrome
**58**	**35**	H	-7025AluI	16519,152,199,	Partial MELAS syndrome
**59**	**35**	H	-7025AluI	16519,152,199,	Partial MELAS syndrome
**60**	**36**	H	-7025AluI	16519,56i,93,	Diabetes Syndrome
**61**	**37**	H	-7025AluI -12308HinfI; -4216NlaIII; +4577NlaIII	16519,73,	clear MELAS syndrome
**62**	**38**	H	-7025AluI	16519,93,95C,	Partial MELAS syndrome
**63**	**39**	H	-7025AluI	93,204,	Partial MELAS syndrome
**64**	**39**	H	-7025AluI	93,204,	Diabetes Syndrome
**65**	**40**	H	-7025AluI	RCRS,	Diabetes Syndrome
**66**	**40**	H	-7025AluI	RCRS,	clear MELAS syndrome
**67**	**41**	R0*	+7025AluI -12308HinfI; -4216NlaIII; +4577NlaIII	16311,	Partial MELAS syndrome
**68**	**42**	R0*	+7025AluI -12308HinfI; -4216NlaIII; +4577NlaIII	16311,16354,	clear MELAS syndrome
**69**	**43**	R0a	+7025AluI +4577NlaIII	16126,16362,16519,64,	clear MELAS syndrome
**70**	**44**	V	+7025AluI -4577NlaIII	16086,16298,72,73,	Diabetes Syndrome
**71**	**45**	V	+7025AluI -4577NlaIII	16129,16298,16311,72,195,	Partial MELAS syndrome
**72**	**46**	V	+7025AluI -4577NlaIII	16162,16298,	clear MELAS syndrome
**73**	**47**	V	+7025AluI -4577NlaIII	16278,16298,72,	clear MELAS syndrome
**74**	**48**	V	+7025AluI -4577NlaIII	16292,16298,72,195,	Diabetes Syndrome
**75**	**49**	V	+7025AluI -4577NlaIII	16298,16301,72,	Partial MELAS syndrome
**76**	**50**	V	+7025AluI -4577NlaIII	16298,16311,72,	clear MELAS syndrome
**77**	**51**	V	+7025AluI -4577NlaIII	16298,72,	clear MELAS syndrome
**78**	**52**	V	+7025AluI -4577NlaIII	16298,72,200,	clear MELAS syndrome
**79**	**53**	V	+7025AluI -4577NlaIII	16298,72,93,	Diabetes Syndrome
**80**	**54**	HV0*	+7025AluI +4577NlaIII	16129,16298,72,195,	Diabetes Syndrome
**81**	**55**	HV0*	+7025AluI +4577NlaIII	16298,72,195,	Diabetes Syndrome
**82**	**56**	T1	+7025AluI +4216NlaIII; +13704BstOI	16126,16163,16186,16189,16294,16304,16519,73,152,195,	clear MELAS syndrome
**83**	**57**	T1	+7025AluI +4216NlaIII; +13704BstOI	16126,16163,16186,16189,16294,16355,16356,16519,73,	clear MELAS syndrome
**84**	**58**	T	+7025AluI +4216NlaIII; +13704BstOI	16126,16147,16294,16296,16297,16304,16519,73,	clear MELAS syndrome
**85**	**59**	T	+7025AluI +4216NlaIII; +13704BstOI	16126,16153,16294,16390,16519,41,73,150,	Diabetes Syndrome
**86**	**60**	T	+7025AluI +4216NlaIII; +13704BstOI	16126,16172,16294,16296,16304,16519,73,	Diabetes Syndrome
**87**	**61**	T	+7025AluI +4216NlaIII; +13704BstOI	16126,16189,16292,16294,16519,73,146,	Diabetes Syndrome
**88**	**62**	T	+7025AluI +4216NlaIII; +13704BstOI	16126,16266,16294,16304,16519,73,	clear MELAS syndrome
**89**	**63**	T	+7025AluI +4216NlaIII; +13704BstOI	16126,16294,16296,16304,16454,16455,16519,73,152,	Diabetes Syndrome
**90**	**64**	T	+7025AluI +4216NlaIII; +13704BstOI	16126,16294,16296,16304,16519,73,	Diabetes Syndrome
**91**	**65**	T	+7025AluI +4216NlaIII; +13704BstOI	16126,16294,16296,16304,16519,73,146,	Partial MELAS syndrome
**92**	**66**	T	+7025AluI +4216NlaIII; +13704BstOI	16126,16294,16296,16519,73,	Partial MELAS syndrome
**93**	**67**	T	+7025AluI +4216NlaIII; +13704BstOI	16126,16294,16304,16519,73,	clear MELAS syndrome
**94**	**68**	T	+7025AluI +4216NlaIII; +13704BstOI	16126,16294,16519,73,146,152,	Partial MELAS syndrome
**95**	**69**	J	+7025AluI +4216NlaIII; -13704BstOI	16069,16126,16234,16235,16519,73,185,188,	Partial MELAS syndrome
**96**	**69**	J	+7025AluI +4216NlaIII; -13704BstOI	16069,16126,16234,16235,16519,73,185,188,	Diabetes Syndrome
**97**	**70**	J	+7025AluI +4216NlaIII; -13704BstOI	16069,16126,16366,16390,16519,73,185,188,	clear MELAS syndrome
**98**	**71**	U2	+7025AluI +12308HinfI	16051,16129,16189,16256,16519,73,152,	Partial MELAS syndrome
**99**	**72**	U2	+7025AluI +12308HinfI	16051,16129C,16183C,16189,16319,16362,16519,73,152,	Diabetes Syndrome
**100**	**73**	U2	+7025AluI +12308HinfI	16051,16129C,16183C,16189,16355,16362,16519,73,152,	Diabetes Syndrome
**101**	**74**	U2	+7025AluI +12308HinfI	16051,16183C,16189,16234,16266,16294,16519,16525,16558,73,152,199,	Diabetes Syndrome
**102**	**75**	U2	+7025AluI +12308HinfI	16051,16183C,16189,16234,16266,16294,16519,16525,73,152,199,	clear MELAS syndrome
**103**	**76**	K	+7025AluI +12308HinfI	16093,16224,16260,16311,16519,44,73,	Partial MELAS syndrome
**104**	**77**	K	+7025AluI +12308HinfI	16093,16224,16311,16319,16519,73,195,	Diabetes Syndrome
**105**	**78**	K	+7025AluI +12308HinfI	16114A,16224,16311,16362,16519,73,195,	Diabetes Syndrome
**106**	**79**	K	+7025AluI +12308HinfI	16223,16224,16234,16311,16519,73,114,	clear MELAS syndrome
**107**	**80**	K	+7025AluI +12308HinfI	16224,16311,16519,64,73,146,152,	Diabetes Syndrome
**108**	**81**	K	+7025AluI +12308HinfI	16224,16311,16519,73,	Diabetes Syndrome
**109**	**82**	K	+7025AluI +12308HinfI	16224,16311,16519,73,146,	clear MELAS syndrome
**110**	**83**	K	+7025AluI +12308HinfI	16224,16311,16519,73,146,152,	Diabetes Syndrome
**111**	**84**	K	+7025AluI +12308HinfI	16224,16311,16519,73,146,185,	Partial MELAS syndrome
**112**	**85**	K	+7025AluI +12308HinfI	16224,16311,16519,73,146,195,	clear MELAS syndrome
**113**	**85**	K	+7025AluI +12308HinfI	16224,16311,16519,73,146,195,	clear MELAS syndrome
**114**	**86**	U5	+7025AluI +12308HinfI	16148,16239,16270,73,150,	Diabetes Syndrome
**115**	**87**	U5	+7025AluI +12308HinfI	16150,16172,16185,16189delT,16270,16274,16311,16465,16519,73,150,152,	Partial MELAS syndrome
**116**	**88**	U5	+7025AluI +12308HinfI	16168,16192,16256,16270,16526,73,	Partial MELAS syndrome
**117**	**89**	U5	+7025AluI +12308HinfI	16189,16192,16270,16398,73,150,	Diabetes Syndrome
**118**	**90**	U5	+7025AluI +12308HinfI	16192,16256,16270,16278,16294,16391,16526,64,73,	Diabetes Syndrome
**119**	**91**	U5	+7025AluI +12308HinfI	16192,16270,16319,16526,73,150,	Partial MELAS syndrome
**120**	**92**	U5	+7025AluI +12308HinfI	16192,16270,16319,73,150,	Diabetes Syndrome
**121**	**93**	U5	+7025AluI +12308HinfI	16256,16270,16399,73,	clear MELAS syndrome
**122**	**94**	U5	+7025AluI +12308HinfI	16270,16296,58,59G,64,73,150,200,	clear MELAS syndrome
**123**	**95**	U4	+7025AluI +12308HinfI	16219,16356,16519,73,195,	clear MELAS syndrome
**124**	**95**	U4	+7025AluI +12308HinfI	16219,16356,16519,73,195,	Diabetes Syndrome
**125**	**95**	U4	+7025AluI +12308HinfI	16219,16356,16519,73,195,	Diabetes Syndrome
**126**	**96**	U4	+7025AluI +12308HinfI	16295,16356,16519,73,195,	Diabetes Syndrome
**127**	**97**	U4	+7025AluI +12308HinfI	16356,16519,73,146,195,	clear MELAS syndrome
**128**	**98**	U4	+7025AluI +12308HinfI	16356,16519,73,195,	clear MELAS syndrome
**129**	**99**	U5	+7025AluI +12308HinfI	16189,16192,16270,16311,16336,55,73,150,	clear MELAS syndrome
**130**	**100**	X	+7025AluI +14465AccI	16129,16189,16223,16278,16290,16362,16519,73,153,195,	clear MELAS syndrome
**131**	**101**	X	+7025AluI +14465AccI	16186,16189,16223,16278,16297,16519,73,153,195,	Diabetes Syndrome
**132**	**102**	X	+7025AluI	16186,16189,16223,16278,16519,73,153,188,195,	Partial MELAS syndrome
**133**	**103**	I	+7025AluI -4529HaeII	16129,16223,16304,16391,16519,73,199,204,	Diabetes Syndrome
**134**	**103**	I	+7025AluI -4529HaeII	16129,16223,16304,16391,16519,73,199,204,	Partial MELAS syndrome
**135**	**104**	N1b	+7025AluI	16145,16176G,16223,16390,16519,73,151,152,	clear MELAS syndrome
**136**	**105**	N1a	+7025AluI	16147A,16172,16223,16248,16355,16519,73,199,204,	clear MELAS syndrome
**137**	**106**	L2	+7025AluI	16129,16189,16192,16223,16274,16278,16294,16309,16390,73,143,146,152,195,	Diabetes Syndrome
**138**	**107**	L2	+7025AluI	16183C,16189,16223,16266,16274,16278,16390,16519,73,152,195,	Partial MELAS syndrome
**139**	**108**	D	+7025AluI -12308HinfI	16221,16223,16291,16362,16390,16519,73,	clear MELAS syndrome
**140**	**109**	W	+7025AluI -8994HaeIII	16223,16280,16292,16519,73,119,189,195,204,	clear MELAS syndrome
**141**	**110**	D	+7025AluI	16223,16295,16362,16519,73,146,199,	Diabetes Syndrome
**142**	**111**	I	+7025AluI -4529HaeII	16223,16311,16391,16519,73,199,204,	clear MELAS syndrome

Partial MELAS syndrome patients are patients who present some, but not all of the typical MELAS symptoms. - Detailed haplogroups [5] and haplotypes

### Analysis of the haplogroups

Each patient's DNA was extracted from blood or muscle using phenol/chloroform or Qiagene^® ^QIAAmp DNA kit. For each patient, the mitochondrial DNA control region was amplified using the following primers: L15832 (light chain, nps 15838–15858) and H408 (heavy chain nps 408–429). The amplification product were purified using ExoSAP-IT^® ^technology. Finally, starting from position 16050, at least 725 pb of the mitochondrial DNA control region were double-strand sequenced using an ABI Prism BigDye Terminator Cycle Sequencing Ready Reaction Kit (Perkin Elmer^®^). The same primers were used for amplification and sequencing, L15832 and H408, together with 2 additional primers: L16200 (light chain, nps 16194–16217) and H263 (heavy chain nps 263–285). Each individual was also tested for the presence of a polymorphism in position 7028 by digestion with the AluI restriction enzyme after amplification with primers L6909 (light chain, nps 6890–6909) and H7115 (heavy chain nps 7115–7131).

**Figure 1:**
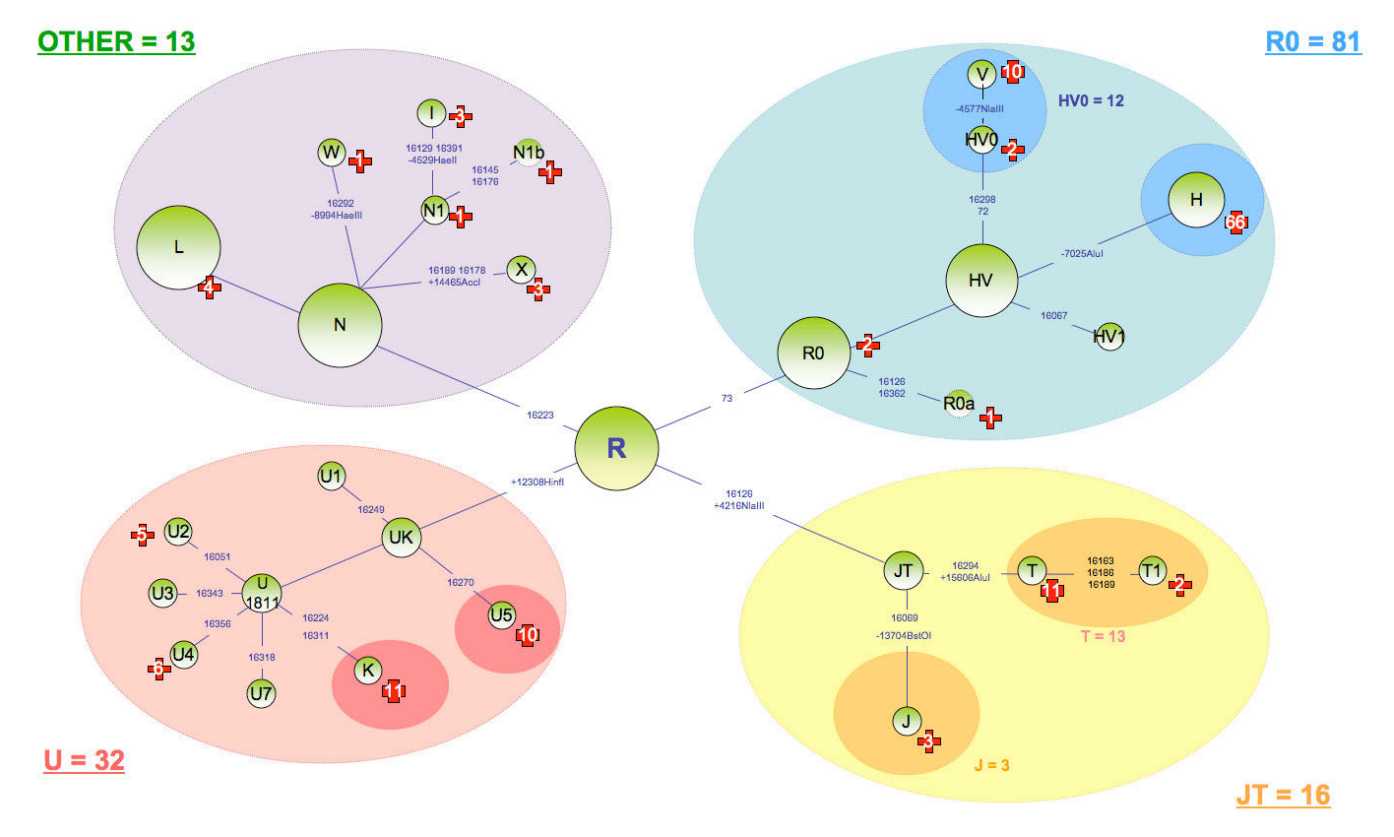
**Phylogenical repartition of the 111 different haplotypes observed in the 142 A3243G carriers**. The phylogenic tree was based on recent total sequencing studies of mitochondrial DNA [33]; the new haplogroup nomenclature is used [5]. The different polymorphisms used for haplogroup determination in this study are indicated in blue. The red crosses indicate the number of individuals per haplogroup.

This made it possible to identify each individual's haplotype and haplogroup markers. The individuals' haplogroup affiliation was determined on the basis of recent studies involving total sequencing of mtDNA [31-34] (Figure 1, Table 1). Affiliations to the main European haplogroups were confirmed by standard RFLP tests (UK: 12308HinfI; JT 4216NlaIII; J: 13704Bsto1; T: 15606AluI; H7025AluI V 4577NlaIII; X:14465AccI; W: 8994HaeIII; I: 4529HaeII) on diagnostic positions for the haplogroups on the mtDNA coding segment (Figure 1). We have used the new haplogroup nomenclature recently proposed by A. Torroni, A. Achilli, V. Macaulay, M. Richards, and HJ. Bandelt [5] in order to classify the patients in haplogroups.

### Control Population and Statistics

The patient's haplotype diversity was compared with a French population sample (210 individuals) [35]. Indeed this study was based on a similar methodology leading to a similar haplotype resolution.

The distribution of carriers of the A3243G mutation among the haplogroups was compared with frequencies observed in a sample of the French population consisting of 1,385 individuals [36]. Each haplogroup was tested as a possible risk factor for developing the pathology, initially against the rest of the population, then versus haplogroup H. Statistical comparisons were carried out on each haplogroup in turn, using standard "Khi 2" methods without correction. The relative risk estimated for each haplogroup was defined by the "odds ratio" method, with a confidence interval calculated by the Miettinen method [37].

## Results

### 1 – Haplotypes and Phylogenetics (Figure 1)

mtDNA D-loop sequencing and RFLP analysis of the coding sequences revealed 111 different haplotypes, among the 142 unrelated carriers of the A3243G mutation (reported on Table 1 and in genbank-National Center for Biotechnology Information with the accession numbers: to EU519009 at EU519150). The ratio of haplotypes to individuals was calculated as 0.78, similar to the value of 0.81 obtained in Dubut et al [35]. The most common haplotype in our population, which is the most common in french population sample, present in 13 individuals (haplotype n°3), differed from the rCRS sequence by a single transition in position 16519. We also found 11 pairs of individuals with the same haplotype and 4 triplets. These haplotypes are also regularly present in the French population sample [35]. Thus the diversity of the population of the A3243G mutation carriers was apparently, qualitatively and quantitatively similar to that of the French population as a whole.

We were also able to classify 139 patients out of 142 in haplogroups with no ambiguity. For the 3 remaining individuals, a set of 4 RFLP tests (12308Hinf1; 4216NlaIII; 7025Alu I; 4577NlaIII) was carried out in order to proceed by elimination.

For individuals 31, 43, and 61, the absence of digestion of site RFLP 7025 indicated that they belonged to haplogroup H, despite the absence of polymorphism A73G on the D-loop, another characteristic of haplogroup H. The analysis of the 4 RFLP of these 3 individuals clearly excluded them from the other European haplogroups. Individual 31 also had polymorphisms 16162 and 16209, known to be associated with an A73G reversion [38]. These three individuals were, therefore, classified in haplogroup H.

The range of haplogroups was extremely wide and included the typically European haplogroups H, R0a, HV0, V, J, T, T1, K, U2, U5, U4, I, X, and W, as well as N1a and N1b, plus two representatives each of African haplogroup L2, and Asian haplogroup D (figure 1). The presence of the last two haplogroups is not unexpected, since African mtDNAs are not uncommon in France and members of Asian super-haplogroup M are sporadically identified in Europe [36].

### 2 – Haplogoup frequencies observed among A3243G patients

The Figure 1 represents the patients distribution on a phylogenetic tree of the European population based on recent total sequencing studies of mitochondrial DNA [33]. The wide distribution of our patients on this tree confirmed our haplotype observations, i.e.: the existence of a great number of mutation events A3243G.

In light of these results (Table 1, Figure 1), we investigated whether there was a difference in distribution among haplogroups of the patient population carrying the A3243G mutation compared to the reference population. We therefore compared the distribution of the 142 A3243G mutation carriers among European haplogroups with the frequencies obtained for 1,385 unrelated French people [36].

For the purposes of statistical comparison, we collected individuals in phylogenetic clusters centered on super-haplogroup R. This point was chosen as it corresponded to the node where the R line, which represents 90 % of the French population, divides off from the rest of the human phylogeny. We then analyzed whether each cluster constituted a risk factor compared to the rest of the population. We tested the 4 largest clusters representative of a phylogenic reality, as well as the haplogroups or smaller sub-haplogroups, so that we could use Khi2 without correction (Table 2).

**Table 2:** Relative risk estimated (Odds ratio: O.R.) of each haplogroup to develop the pathology against the rest of the population.

Haplogroup	3243 carriers	French Population	O.R.	P. value	C.I. at 95%
**R0**	**57.0%**	**52.4%**	**1.21**	**0.29**	**0.85–1.71**
> H	46.5%	45.6%	1.04	0.84	0.73–1.46
> HV0	8.5%	4.8%	1.83	0.06	0.96–3.47
**JT**	**11.3%**	**16.2%**	**0.66**	**0.12**	**0.38–1.13**
> T	9.2%	8.5%	1.08	0.79	0.59–1.98
> J	2.1%	7.7%	0.26	0.01	0.08–0.83
**UK**	**22.5%**	**21.9%**	**1.04**	**0.86**	**0.69–1.57**
> U5	7.0%	8.3%	0.84	0.60	0.43–1.64
> U1811	15.5%	13.5%	1.17	0.51	0.73–1.90
>> K	7.7%	8.7%	0.88	0.70	0.46–1.68
**OTHER**	**9.2%**	**9.5%**	**0.96**	**0.89**	**0.53–1.75**

The new haplogroup nomenclature is used (Torroni et al. 2006). C.I.: Confidence Interval.

#### Super-haplogroup R0 (formerly PreHV*)

Haplogroup R0 included 81 patients (Figure 1), i.e. 57.0% of the A3243G mutation carriers. The slight overrepresentation (OR: 1.21, C.I. 95%: 0.85–1.71) of this haplogroup was not significant (p = 0.29) (Table 1). To obtain more detailed results, we applied the same tests to all sufficiently large sub-clusters of R0, i.e. haplogroups H and HV0. This revealed an overrepresentation of haplogroup HV0 (formerly pre-V) (OR: 1.83, C.I. 95%: 0.96–3.47), however this result is not clearly significant (p = 0.06). Individuals in haplogroup HV0 had 12 distinct haplotypes, which would seem to exclude the possibility that overexpression of this haplogroup was due to a single mutation event. The frequency of haplogroup H did not seem significantly different from that observed in the control population (p = 0.73). In view of the lack of data on the proportions of H sub-haplogroups in the French population, as well as the considerable variation in the frequency of sub-haplogroups in Western Europe [32], we decided not to subdivide this haplogroup.

#### Super-haplogroup UK

22.5% of our patients were identified as belonging to haplogroup UK, which was not significantly different from the French population (p = 0.86, OR: 1.04, C.I. 95%: 0.69–1.57). As in the case of haplogroup R0, we tested for significant differences in the sub-clusters of this haplogroup, however, there was no significant difference for sub-clusters U5 (p = 0.60), U1811 (p = 0.51), or K (p = 0.70).

#### Super-haplogroup OTHER

All the patients who were not in super-cluster R were classified in super-haplogroup OTHER. This was, therefore, a broadly paraphyletic group, including haplogroups: N1, I, W, X, M, and L. 13 patients were classified in this haplogroup. The comparison of this cluster with the "OTHER" group of the reference study has detected no significant difference (p = 0.89, OR = 0.96, C.I. 95%: 0.53–1.75)

#### Super- haplogroup JT

Haplogroup JT represented 11.3% of our study population, and was, therefore, slightly underrepresented (p = 0.12, OR = 0.66, C.I. 95%: 0.38–1.13). J haplogroup, with only 3 individuals, seemed very significantly underrepresented among our patients (p = 0.01, OR = 0.26, C.I. 95%: 0.08–0.83). Moreover, these 3 patients can be caracterised by a later onset (between 20–40 years) but with no specific symptomatology, toward the MELAS patients. There was not, apparently, any significant difference in haplogroup T (p = 0.79, OR = 1.08, C.I. 95%: 0.59–1.98). The underrepresentation of Haplogroup JT was apparently, simply due to underrepresentation of J.

#### Using haplogroup H as a reference

We also tested whether each cluster constituted a risk factor in comparison to a single reference haplogroup. As presented by van der Walt et al. [39] using a reference haplogroup is more homogeneous than the sum of the various haplogroups. In addition, it was possible to compare each haplogroup variation between the control population and our patients with the variation in the same reference haplogroup. The choice of haplogroup H as a reference was based on the fact that it did not seem to vary significantly between the control and patient populations. In addition, the haplogroup H was the most widely represented in the French population. The results of this analysis (Table 3) confirmed the absence of variation for clusters OTHER, UK, and T (p > 0.6) and emphasized the underexpression of J (p = 0.02, OR = 0.27, C.I. 95%: 0.08–0.87). Moreover, the overexpression of HV0 is no more observed (p = 0.10, OR = 1.73, C.I. 95%: 0.89–3.36).

**Table 3:** Relative risk estimated (Odds ratio: O.R.) of each haplogroup to develop the pathology against the H haplogroup.

Haplogoup	3243 carriers	French Population	O.R	P. value	C.I. at 95%
**R0**	**57.0%**	**52.4%**			
> H	46.5%	45.6%			
> HV0	8.5%	4.8%	1.73	0.10	0.89–3.36
**JT**	**11.3%**	**16.2%**	**0.68**	**0.18**	**0.39–1.20**
> T	9.2%	8.5%	1.06	0.86	0.56–1.98
> J	2.1%	7.7%	0.27	0.02	0.08–0.87
**UK**	**22.5%**	**21.9%**	**1.01**	**0.97**	**0.65–1.57**
> U5	7.0%	8.3%	0.83	0.60	0.42–1.67
> U1811	15.5%	13.5%	1.13	0.65	0.68–1.87
>> K	7.7%	8.7%	0.87	0.69	0.45–1.70
**OTHER**	**9.2%**	**9.5%**	**0.95**	**0.86**	**0.51–1.76**

The new haplogroup nomenclature is used (Torroni et al. 2006). C.I.: Confidence Interval.

## Discussion

The A3243G mutation, located on the tRNALeu gene (UUR) [16], is one of the most common mitochondrial DNA mutations worlwide. The phenotypes associated with this disease are highly variable and heterogeneous. The impact of mitochondrial genetic background is increasingly being considered, in explanation of the phenotype variability common to pathogenic mitochondrial mutations. Each phenotype would, therefore, consist of one main pathogenic mutation, associated with secondary mutations, which modulate the expression of the main mutation. Secondary mutations do not necessarily have any impact on the phenotype of an individual who does not carry the main mutation and are, therefore, generally considered to be polymorphisms.

Progress in mitochondrial phylogeny has made it possible to classify mitochondrial genotypes (haplotypes) in monophyletic lines (haplogroups). Thus, two mitochondrial haplotypes in the same haplogroup will have the same phylogenetic background and, therefore, share a certain number of polymorphisms [5].

To determine whether these polymorphisms have an impact on pathogenic mitochondrial mutations, previous studies investigated the statistical correlations between pathogenic mutations and mitochondrial haplogroups [10]. Most of these studies have been carried out with very small cohorts. The largest cohort of bearers of the same mutation from a single population to date consisted of 67 Italian patients with mutation 11778 [12]. Previous European research into the A3243G mutation analyzed data from 35 Spains [29], 11 Finns [40], and 7 Dutch [15]. As suggested by Samuels et al [30]; in order to make informative statistical analysis, this type of study requires a maximum number of patient carrying the same pathogenic mutation within the same population. To overcome this difficulty, the present study included almost all the patients diagnosed by the French mitochondrial pathology network in recent years. Besides including a large number of unrelated patients (142), this made it possible to study a population of patient spread all over the French territory. Furthermore, the French health care system provides access to treatment to all individuals living in France, so our results are not biased by the possible recruitment of specific sub-classes of the population.

### Appearance of the A3243G mutation

The present study showed that the A3243G mutation carriers were distributed among all the haplogroups commonly found in the European population. This may suggested that the A3243G mutation appeared many times, as hypothesized in previous studies [15,29,40]. Moreover, the genetic diversity of our population is quantitatively and qualitatively similar to that of the French population. The population of patients carrying the mutation A3423G did not, therefore, seem to have been affected by founding events, but, on the contrary, to be due to a large number of independent mutation events.

In a similar analysis of LHON pathology, it was suggested that the number of independent mutation events could be assimilated to the number of different haplotypes [12]. Indeed, the haplotypes found in the patient population were found at random in a sample of the reference population. Similarly, the haplotypes found in our patient population were also present in our reference sample. The presence of these haplotypes in the reference population indicated that most of the 3243 mutation events had appeared independently in haplotypes existing in the healthy population.

Furthermore, the most common haplotype in our population (n°3) was associated with separate H sub-haplogroups [38], which are also very common in the French population. Therefore, it is highly probable that the patients who had this same haplotype resulted from several independent mutation events. Following analysis of 111 haplotypes, we therefore suggest that at least 111 A3243G transition events have occurred recently in the French population.

Antonio Torroni et al [29] suggested classifying the A3243G mutation as a "severely deleterious" mutation, according to the Douglas Wallace classification [41]. This category includes heteroplasmic mutations that produce a very handicapping phenotype, transmitted only over very few generations. Our results confirmed this classification of the A3243G mutation. In this "severely deleterious" mutation scenario, individuals affected by the A3243G mutation on 16.3/100,000 [42] would seem to result mainly from recurrent mutation phenomena, rather than founding effects associated with mutation transmission and propagation phenomena.

This large number of independent mutation events is apparently linked to the existence of a mutation hot spot on tRNA leucine 1 [43,44]. Indeed, 24 "probable" pathogenic mutations, including 7 "confirmed", have been identified on the Mitomap Website for tRNA leucine 1 (where the A3243G mutation is located), which is 70 base pairs long. This level of mutation seems particularly high compared with tRNA leucine 2, where only 5 "probable" pathogenic mutations have been identified, including only 1 "confirmed". tRNAleu 1 and tRNAleu 2 have approximately the same length and the same function, so the higher mutation rate of tRNAleu 1 may be due to its second function. Indeed the sequence of tRNAleu 1 is implied with the mTERF factor in a mitochondrial DNA transcription termination zone; furthermore, this transcription mechanism has been shown to involve the formation of two loops [45]. It is, therefore, possible that this mechanism blocking mitochondrial RNA-polymerase (mtRPOL) activity is also able to reduce the accuracy of DNA-polymerase γ.

Taken together, our findings demonstrated the absence of a decrease in diversity within the patient haplogroups. This indicated that mitochondrial genetic background had no impact on mutation events and did not, therefore, affect the mutation rate of position A3243G.

### Impact of mitochondrial genetic background

Although genetic background does not affect the frequency of mutation 3243, it may modulate its expression. We compared the distribution of 142 unrelated French patients affected by the A3243G mutation among European haplogroups, with that of the French population sample of 1,385 individuals described by Richard et al. The distribution of the A3243G mutation carriers among European haplogroups revealed a significant difference in J haplogroup compared to the French population. These differences apparently demonstrated a link between haplogroups and the A3243G mutation and, therefore, that mitochondrial genetic background had an impact on the A3243G mutation. These findings are apparently in contradiction with a previous study involving 35 Spanish patients [29], however, the lack of differences in the Spanish cohort may probably due to the small size of the samples.

The impact of genetic background on mutation T14484C, which is related to LHON pathology, was more striking, as 75% of the carriers of this mutation belonged to J haplogroup. However, the significant presence of the A3243G mutation in all European haplogroups may be explained by a combination of 3 factors: (i) a high mutation rate; (ii), the absence of polymorphisms related to European haplogroups located directly on tRNAleu 1, which makes it impossible to compensate or aggravate directly this mutation; (iii) tRNA function itself, necessary for the traduction of all the mitochondrial genes, which makes it impossible to have a global compensation by the presence of a polymorphism located on a single gene.

J haplogroup was markedly underrepresented among the A3243G mutation carriers. There are different possibilities to explain this observation. It might be possible that (i) this combination produced a phenotype not commonly considered to be from mitochondrial origin. Then, these patients would not be studied for mtDNA mutations and they would form part of other patient populations.

J haplogroup could also (ii) be a protective genetic background. However, this hypothesis is relatively improbable for several reasons. First, tRNA leucine is not affected by any polymorphisms linked with J haplogroup, so it is difficult to hypothesize compensation in terms of tRNA leucine conformation. Second, as explained in the previous paragraph, is highly improbable that the related polymorphisms of J are able to produce a global compensation of the A3243G mutation. Finally, if the A3243G mutation were attenuated in J haplogroup, due to its high mutation rate, it could be identified in the sample of healthy fully-sequenced individuals belonging to J haplogroup (Mitomap). However, if we did not find it, the hundred of mtDNA J published sequences that we have now are not enough to find this combination on the general population. For example, if the A3243G frequency was 16 out of 100,000 individuals (1 out of 6,250) and the population frequency of the haplogroup J is around 10%, 62,500 individuals should be sequenced to find the mutation in the general population.

**Figure 2:**
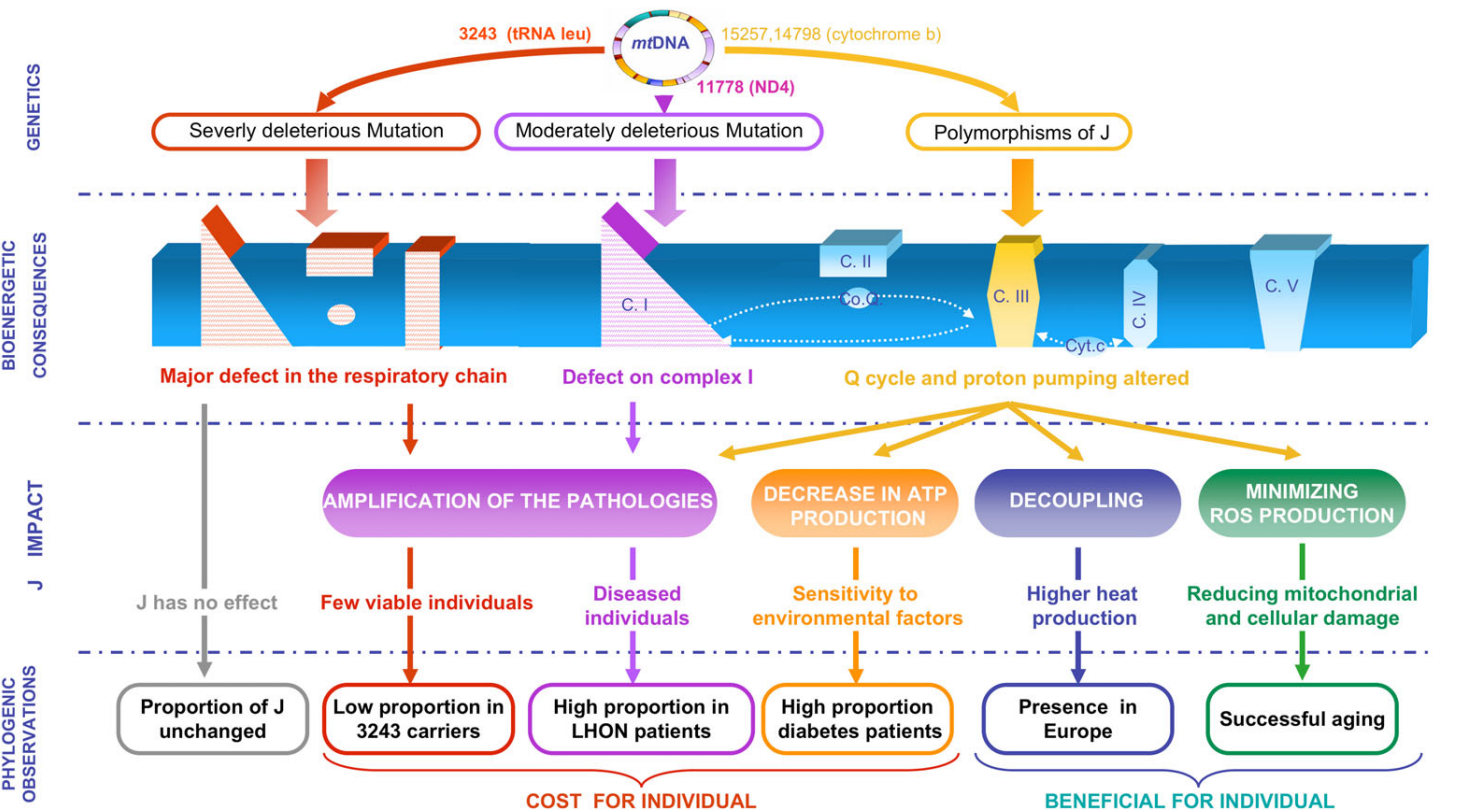
**Hypothetical mechanism of the "J paradox"**. The impact of J polymorphisms can differed toward the mitochondrial environment. This figure summarizes these impacts on the OXPHOS level, their implications on an individual level, and their impact on the distribution of populations studied within the phylogeny.

In light of these facts, we put forward a third hypothesis, (iii): the association of J haplogroup and A3243G is life-threatening, and therefore lethal to the embryo or germ line. This hypothesis agrees more closely with previous findings that associate J with a number of diseases, such as LHON [11-13], diabetes [46], and optic neuritis [47] (Figure 2). The life-threatening action of J haplogroup has been suggested to have resulted from an accumulation of non-synonymous polymorphisms on the cytochrome *b *gene [12]. These complex III damages affects, more specifically, the CoQ binding sites, thus upsetting the proton pump Q cycle and, finally, Oxidative Phosphorylation (OXPHOS) coupling (Figure 2) [2,9]. Some authors suggested that this OXPHOS uncoupling characteristic was an ancient mitochondrial adaptation to the cold, as it promoted heat production [8]. The same authors suggested that this uncoupling would reduce ROS production, leading to a lower gradual mitochondria degradation observed in aging. This could explain the overrepresentation of J among populations of people over 100 years [48,49].

Taken together, these points emphasize the paradox of haplogroup J, which may promote the expression of mitochondrial mutations in certain individuals, but also be beneficial for others. Figure 2 summarizes what we named "J paradox", showing the impact of polymorphisms in J haplogroup on the OXPHOS level, their implications on an individual level, and their impact on the distribution of populations studied within the phylogeny. This paradox, already described by D.C. Wallace, E. Ruiz-Pesini, and A. Torroni (importance of cytochrome *b*, relationship between long lifespan, adaptation, and predisposition for diseases), clearly indicates that the distribution of J haplogroup in a patient population could vary considerably and, above all, that this distribution could provide information on the type of mutation and the mechanism involved.

Thus, the effect of a slight decrease of the mitochondrial activity (from a genetic or environmental origin) would be amplified by polymorphisms related to J, resulting in a high proportion of J haplogroup in the patient population. In the case of a more deleterious mutation (as presented here), amplification of the pathology resulted in a lower viability of J-3243 patients, and therefore a low proportion of J among patients with this severe pathology.

## Conclusion

Taken together, these findings show that the A3243G mutation has appeared independently on many occasions in the French population, thus confirming the presence of a mutation hot spot on tRNA leucine 1. Inter-haplogroup differences in mitochondrial genetic backgrounds apparently had no impact on the mutation rate of position 3243. However, there is a link between the haplogroup, especially J haplogroup, and expression of the mutation. Our study confirmed the susceptibility of this haplogroup to the expression of pathologies of mitochondrial origin. If the Western Eurasian haplogroup J appears to be associated to the prevalence of the A3243G mutation, it is highly provable that other haplogroups from other regions of the world could be associated. In this sense, two different studies found, first, a higher frequency of the 9 bp deletion in Taiwan MELAS patients [26] and, second, an overrepresentation of the T16189C polymorphism in patients with the A3243G mutation [50] Both markers define mtDNA haplogroup B.

These results highlight the importance of analyzing mitochondrial genetic background when studying mitochondrial diseases. Indeed, patients' haplogroups could be a part of the diagnostic procedure for mitochondrial diseases, in order to obtain a clearer understanding of their modulation mechanisms, and therefore adjust the diagnosis.

## Competing interests

The authors declare that they have no competing interests.

## Authors' contributions

DP and TL have conceived of the study and performed the determination of the haplogroup. PM and DT have participated in its design and coordination and helped to draft the manuscript. Screening for mitochondrial diseases was carried out by the French mitochondrial disease network: Angers (PA and PR), Bordeaux (ML MN, RR and CR), Caen (SA), Grenoble (CB), Lyon (BMC, CG), Paris (AR, DF, CBC). BA, EP and MC have provided technical support. All authors read and approved the final manuscript.

## Pre-publication history

The pre-publication history for this paper can be accessed here:


